# Preparation and Luminescence Property Study of Red-Emitting Na_3.6_Y_1.8_(PO_4_)_3_:Eu^3+^,Li^+^/K^+^ Phosphors with Excellent Thermal Stability for Light-Conversion Application

**DOI:** 10.3390/nano14211721

**Published:** 2024-10-29

**Authors:** Qi You, Xuan Zhou, Chengxiang Yang, Mu Liu, Wei Liu, Jinkai Li, Xuchuan Jiang

**Affiliations:** 1 Institute for Smart Materials & Engineering, University of Jinan, No. 336 Nanxinzhuang West Road, Jinan 250022, China; ism_youq@ujn.edu.cn (Q.Y.); 202211100036@stu.ujn.edu.cn (C.Y.); 2 Shandong Jigang Luna New Material Technology Co., Ltd., 1819-1, Qingyuan Street, Bucun Subdistrict, Zhangqiu District, Jinan 250200, China; 202121100212@stu.ujn.edu.cn (X.Z.); 202121201116@stu.ujn.edu.cn (M.L.); 3 School of Materials Science and Engineering, University of Jinan, No. 336 Nanxinzhuang West Road, Jinan 250022, China; mse_lijk@ujn.edu.cn

**Keywords:** alkali metal ion substitution, luminescence enhancement, red-emitting phosphor, plant growth

## Abstract

A series of red-emitting phosphors, Na_3.6_Y_1.8−x_(PO_4_)_3_:xEu^3+^, have been synthesized by a high-temperature solid-phase method. The impact of the partial Li^+^/K^+^ ion substitution on the crystal structure and photoluminescence (PL) performance of Na_3.6_Y_1.05_(PO_4_)_3_:0.75Eu^3+^ phosphor have been investigated. Various techniques have been used for characterization of the as-obtained materials. X-ray diffraction (XRD) analysis was utilized to confirm the composites of these samples, and the morphology and element distribution were examined by scanning electron microscope (SEM) and transmission electron microscope (TEM). This study found that the developed Na_3.6_Y_1.8−x_(PO_4_)_3_:xEu^3+^ phosphors exhibited a prominent emission peak at ~620 nm when excited at 393 nm, which corresponded to ^5^D_0_ → ^7^F_2_ transitions of Eu^3+^ ions. Furthermore, the robust emission peak at ~705 nm (^5^D_0_ → ^7^F_4_) of these phosphors enables a better match with plant pigment absorption. Beyond that, the partial substitution of Li^+^/K^+^ ions probably changed the crystal structure, and reduces the symmetry around Eu^3+^, leading to significantly enhanced luminous intensities by 23.24% and 18.29%, with the highest quantum yields (QYs) reaching 99.85% and 96.29%, respectively. Additionally, the prepared phosphors show non-thermal quenching and superior thermal stability at elevated temperatures from 298 to 473 K. These findings and results suggest that Li⁺/K⁺-substituted Na_3.6_Y_1.05_(PO₄)₃:0.75Eu^3^⁺ phosphors can serve as promising red-emitting phosphors for plant lighting applications.

## 1. Introduction

Natural factors such as storms, floods, droughts, pests and diseases pose significant challenges to agricultural production [1,2,3]. Indoor plant cultivation, supported by artificial plant growth lighting, offers a viable solution by providing a controlled environment conducive to stable plant growth, thereby enhancing crop yield and quality [4,5,6]. However, conventional light sources, including high-pressure sodium lamps, incandescent lamps and fluorescent lamps, have numerous limitations, including high energy consumption, short lifetime and spectrum mismatch [7,8]. In contrast, light-emitting diodes (LEDs) designed specifically for plant growth have gained considerable attention due to their energy efficiency, extended operational lifespan, environmental sustainability, and ability to deliver tunable spectral outputs. This fact notwithstanding, a key need remains: the precise tailoring of LED spectra to cover the critical wavelengths of blue, red, and deep red light, essential for advancing agricultural practices [9,10,11]. The purpose of this work is to improve the PL properties of red-emitting phosphors for plant growth LEDs and consequently boost their efficacy in promoting plant development.

To synthesize advanced phosphors with superior luminous properties for plant growth LEDs, a key step is to identify an appropriate host material. Rare earth-doped phosphates have garnered significant attention due to their wide band gap, excellent luminescent properties, rigid structure, good thermal stability, and high quantum efficiency [12,13,14]. Among them, numerous NASICON phosphates with rare earth ions, such as Na_3_Sc_2_(PO_4_)_3_ and Na_3.6_Y_1.8_(PO_4_)_3_, have been shown to be promising substrates for luminescent materials. Several synthesized phosphors, including Na_3_Sc_2_(PO_4_)_3_:Eu^3+^ [15], Na_3.6_Y_1.8_(PO_4_)_3_:Ce^3+^,Tb^3+^ [16] and Na_3.6_Y_1.8_(PO_4_)_3_:Dy^3+^[17], have demonstrated outstanding performance. For example, Zhang et al. synthesized Na_3.6_Y_1.8_(PO_4_)_3_:Eu^3+^ red phosphors and found that the luminescence intensity remained stable up to 374 K, exhibiting non-thermal quenching and excellent thermal stability [18]. Despite these advancements, previous studies on Na_3.6_Y_1.8_(PO_4_)_3_:Eu^3+^ phosphors have not thoroughly investigated the detailed luminescent properties of Eu^3+^, and further improvement in performance is required for practical applications. Eu^3+^ ions are known for their stable chemical characteristics, ability to be excited by near-ultraviolet light (n-UV), and prominent emission peaks in the red region, making them widely used as activators for red phosphors. A well-known approach for enhancing luminescent performance involves the partial or complete substitution of cations in the host lattice, which can alter the crystal structure and modify the local crystal field and coordination environment surrounding the luminescent center ions [19,20,21]. Among these strategies, alkali metal cation substitution is feasible and has been widely deployed [22,23]. For instance, In K_2_MgGeO_4_:Bi^3+^, alkali metal cation substitution has been employed to achieve luminescence tuning, enhance thermal stability, and develop an ultra-wideband single-phase white phosphor [24]. Furthermore, the substitution of Na^+^ ions with Li^+^/K^+^ in NaLaMgWO_6_:Eu^3+^ red phosphors significantly enhances luminescence intensity [25]. Therefore, it is necessary to investigate the effects of alkali metal ion substitution in Na_3.6_Y_1.8_(PO_4_)_3_:Eu^3+^ phosphors to further optimize their luminescence performance.

In this study, a series of Na_3.6_Y_1.8_(PO_4_)_3_:Eu^3+^,Li^+^/K^+^ phosphors were synthesized using a high-temperature solid-state reaction method. The prepared phosphors were characterized through various techniques, including PL excitation and emission spectra, lifetime measurements, XRD and SEM. Additionally, the effects of different alkali metal ion substitution on crystal morphology and luminescence properties of Na_3.6_Y_1.8_(PO_4_)_3_:Eu^3+^ phosphor were systematically investigated. The substitution of Na^+^ by Li^+^ and K^+^ ions in Na_3.6_Y_1.8_(PO_4_)_3_ leads to changes in crystal field and symmetry, which further affected the luminescent properties. Furthermore, the phosphor with the highest luminescence performance was integrated with a near-ultraviolet (n-UV) chip to fabricate a red LED. Our findings indicate that the luminescence properties can be effectively enhanced through alkali metal ion substitution, suggesting their promising potential for application in plant lighting applications.

## 2. Experimental Section

### 2.1. Sample Synthesis

All these samples were synthesized with a high-temperature solid-state reaction method. First, stoichiometric ratios were used to weigh the chemicals Na_2_HPO_4_, NaH_2_PO_4_, LiH_2_PO_4_, KH_2_PO_4_, Eu_2_O_3_ and Y_2_O_3_ (Aladdin, Shanghai, China). Second, these materials were ground and mixed evenly after that. Then, the mixtures were heated for 5 h in the air to 1373–1773 K. Eventually, all of the as-synthesized materials were chilled to room temperature and ground into a powder once more in preparation for additional research.

(1) $${2.4Na\left(Li,K\right)H}_{2}{PO}_{4}+0.6{Na}_{2}HPO_{4}+0.9Y_{2}O_{3}=Na{\left(Li,K\right)}_{3.6}Y_{1.8}{\left(PO_{4}\right)}_{3}+2.7H_{2}O$$

### 2.2. Characterization

The crystalline phase information was collected through X-ray diffraction (XRD, Model SmartLabSE, Rigaku, Tokyo, Japan) using a Cu Kα radiation (λ = 1.5406 Å) at the conditions of 40 kV and 40 mA at room temperature from 10° to 70° and a velocity of 5°/min in the range of 2θ.

The morphology and element mapping were determined by scanning electron microscopy (SEM, Regulus 8100, Hitachi, Tokyo, Japan), transmission electron microscopy (TEM, JEOL, JEM-2010, Tokyo, Japan) and high-resolution transmission electron microscope (HRTEM, JEM-2100F, JEOL, Tokyo, Japan) equipped with energy dispersive X-ray spectroscopy.

A UV–vis–NIR spectrophotometer (UV-3600 Plus, SHIMADZU, Kyoto, Japan) was implemented to measure the diffuse reflection spectra (DRS).

The PL excitation and emission spectra, QY and decay curves were obtained by an FLS1000 photoluminescence spectrometer (Edinburgh, Livingston, UK). The instrument software was also utilized for gathering the relevant QY calculations. Applying the same device, which was equipped with a self-controlled heating platform that worked between 298 and 473 K, temperature-dependent PL emission spectra were measured and recorded.

## 3. Results and Discussion

### 3.1. XRD Analysis

XRD was employed to analyze the phase structure of the Na_3.6_Y_1.8_(PO_4_)_3_ crystal calcined at various temperatures, as depicted in Figure S1a. When the calcination temperature is higher than 1473 K, all diffraction peaks exhibited excellent agreement with the cubic phase standard cards (PDF#47-0972) possessing a Pm-3m space group. No significant impurity peaks were detected, indicating that the Na_3.6_Y_1.8_(PO_4_)_3_ host remained in a pure phase across the temperature range. Additionally, as the calcination temperature increases, the baseline becomes smoother, and the diffraction peak intensity increases with reduced half-peak breadth. Such changes reflect an increase in crystallinity and coarser grit formation, as well as higher crystallinity and grain coarsening [26]. However, at 1773 K, the samples began to melt, making 1573 K the optimal calcination temperature for this study.

A series of Eu^3+^-doped phosphors were synthesized, and the XRD patterns of Na_3.6_Y_1.8−x_(PO_4_)_3_:xEu^3+^ phosphor with varying Eu^3+^ content are shown in Figure S1b. As Eu^3+^ doping increased, the diffraction peak of the prepared sample continued to align well with the standard card PDF#47-0972 without impurity peaks, suggesting that Eu^3+^ doping does not significantly distort or damage the lattice structure of Na_3.6_Y_1.8_(PO_4_)_3_. Furthermore, it can be observed that as the concentration of Eu^3+^ increases, the strongest diffraction peak gradually shifts to the lower degree, probably due to the substitution of smaller Y^3+^ ions (0.9 Å) with larger Eu^3+^ ions (0.947 Å) in the host lattice.

**Figure 1:**
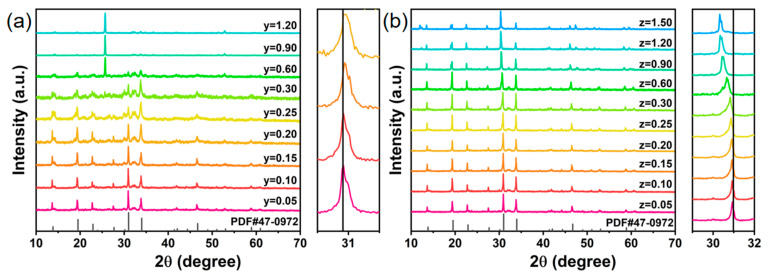
Powder XRD patterns of (**a**) Na_3.6-y_Li_y_Y_1.05_(PO_4_)_3_:0.75Eu^3+^ (0.05 ≤ y ≤ 1.20) and (**b**) Na_3.6-z_K_z_Y_1.05_(PO_4_)_3_:0.75Eu^3+^ (0.05 ≤ z ≤ 1.50) phosphor.

To assess the solid solubility of Li^+^/K^+^ ions in Na_3.6_Y_1.05_(PO_4_)_3_:0.75Eu^3+^ phosphors, XRD patterns of all Li^+^/K^+^ substituted samples are displayed in Figure 1. For the concentration of Li^+^ ranges from 0.05 to 0.20, the diffraction peaks of the phosphors match well with the standard cards, confirming that appropriate concentrations of Li^+^ have minimal impact on the host structure. As Li^+^ concentration increases, the diffraction peaks shift toward higher angles, indicative of lattice contraction due to the substitution of Na^+^ by smaller Li^+^ ions. However, when Li^+^ concentration reaches between 0.25 and 1.20, an abnormality is observed in the main diffraction peak of the samples, likely due to the formation of unphased solid solutions caused by host melting. In contrast, the XRD pattern of K^+^-substituted samples showed no impurity or secondary phase formation, indicating that the Na_3.6-z_K_z_Y_1.05_(PO_4_)_3_:0.75Eu^3+^ maintained the lattice structure across the entire range of K^+^ doping (0–1.50). And the diffraction peaks shift toward a lower angle, indicating that the larger K^+^ ions occupy the Na^+^ site, leading to lattice expansion.

### 3.2. SEM and TEM Analysis

**Figure 2:**
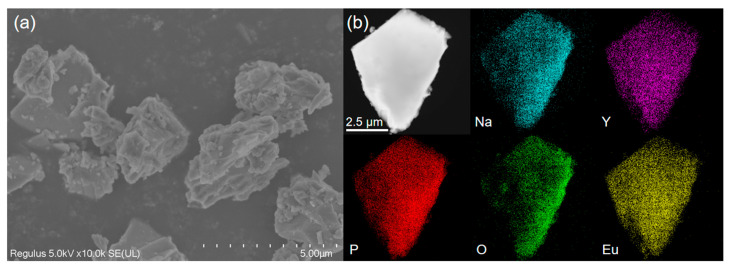
(**a**) SEM image, (**b**) TEM and EDS elemental mappings (Na, Y, P, O and Eu) images of Na_3.6_Y_1.05_(PO_4_)_3_:0.75Eu^3+^ sample.

The SEM images in Figure 2 and Figure S2 show the morphologies of Na_3.6_Y_1.05_(PO_4_)_3_:0.75Eu^3+^, host, Na_3.5_Li_0.1_Y_1.05_(PO_4_)_3_:0.75Eu^3+^ and Na_3.6_K_0.2_Y_1.05_(PO_4_)_3_: 0.75Eu^3+^ samples prepared at 1573 K, respectively. It can be seen that the particles exhibit irregular shapes with an average size of approximately 5 μm, displaying both agglomeration and non-uniform size distribution. In addition, the surface of the particles appear rough, with noticeable cross-sectional features likely resulting from the re-grinding process after the sample was subjected to heating.

To further examine the microstructure, elemental composition and distribution of the synthesized phosphors, TEM and EDS elemental analysis were carried out on representative Na_3.6_Y_1.05_(PO_4_)_3_:0.75Eu^3+^, Na_3.5_Li_0.1_Y_1.05_(PO_4_)_3_:0.75Eu^3+^ and Na_3.6_K_0.2_Y_1.05_(PO_4_)_3_:0.75Eu^3+^ samples, and the results are shown in Figure 2b and Figure S3. The element mapping images of the phosphors reveal K, Na, Y, P, O and Eu elements in different colors, confirming that these elements are uniformly distributed throughout the sample. These findings further validate the successful doping of Eu^3+^ and substitution of Li^+^/K^+^ ions without the introduction of any impurities.

### 3.3. DRS Analysis

**Figure 3:**
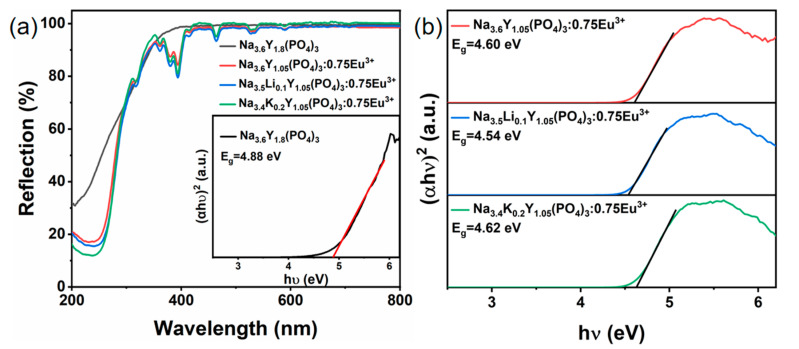
Diffuse reflectance spectra (**a**) and the band gap (**b**) of Na_3.6_Y_1.8_(PO_4_)_3_ (black line, the inset of Figure 3a), Na_3.6_Y_1.05_(PO_4_)_3_:0.75Eu^3+^ (red line), Na_3.5_Li_0.1_Y_1.05_(PO_4_)_3_:0.75Eu^3+^ (green line) and Na_3.6_K_0.2_Y_1.05_(PO_4_)_3_:0.75Eu^3+^ (blue line).

The diffuse reflectance spectra (DRS) of several representative samples are shown in Figure 3a. The DRS of the Na_3.6_Y_1.8_(PO_4_)_3_ host exhibits a broad absorption band extending from 200 to 400 nm, which can mainly be explained by the characteristic light absorption transition from the valence band (VB) to the conduction band (CB) of the host [27]. Upon the introduction of the activator Eu^3+^ into the host, the charge transfer band (CTB) of O^2−^-Eu^3+^ is responsible for the enhanced absorption area at 200–350 nm. The most prominent absorption peaks of Eu^3+^ 4f-4f are found at 393 nm, originating from the ^7^F_0_ → ^5^L_6_ transitions of Eu^3+^ ions [28,29]. Furthermore, after a small amount of alkali metal ion substitution, the DRS of these samples are basically consistent with that of Na_3.6_Y_1.05_(PO_4_)_3_:0.75Eu^3+^. The optical band gap (*E_g_*) can be calculated using the following formula based on the UV–vis absorption data [30,31]:

(2) $${\left(\alpha h\nu \right)}^{n}=B\left(h\nu -E_{g}\right)$$

(3) $$\alpha =\frac{1-R^{2}}{2R}$$

where *h* is Planck’s constant, *ν* is frequency, *B* is a constant, and *n* can be 1/2 and 2, corresponding to indirect transition and direct transition, respectively. The absorption coefficient *α* can be obtained through Equation (3), *R* is the observed reflectance in the DRS. The *E_g_* of Na_3.6_Y_1.8_(PO_4_)_3_ can be determined to be 4.88 eV, as shown in the inset of Figure 3a, indicating that Na_3.6_Y_1.8_(PO_4_)_3_ is suitable to function as the host of luminous materials [32,33]. In addition, it can be observed that *E_g_* is about 4.60, 4.54, and 4.62 eV for Na_3.6_Y_1.05_(PO_4_)_3_:0.75Eu^3+^, Na_3.5_Li_0.1_Y_1.05_(PO_4_)_3_:0.75Eu^3+^ and Na_3.6_K_0.2_Y_1.05_(PO_4_)_3_:0.75Eu^3+^ samples in Figure 3b. Following doping of Eu^3+^, a slight decrease in *E_g_* has been noticed, which is caused by the Burstein–Moss effect [34]. Notably, the substitution of Li^+^/K^+^ culminates in a minor modification of the band gaps, suggesting that these ions entered the host lattice and affected the band structure.

### 3.4. Luminescence Properties of Na_3.6_Y_1.8_(PO_4_)_3_:Eu^3+^ Phosphors

**Figure 4:**
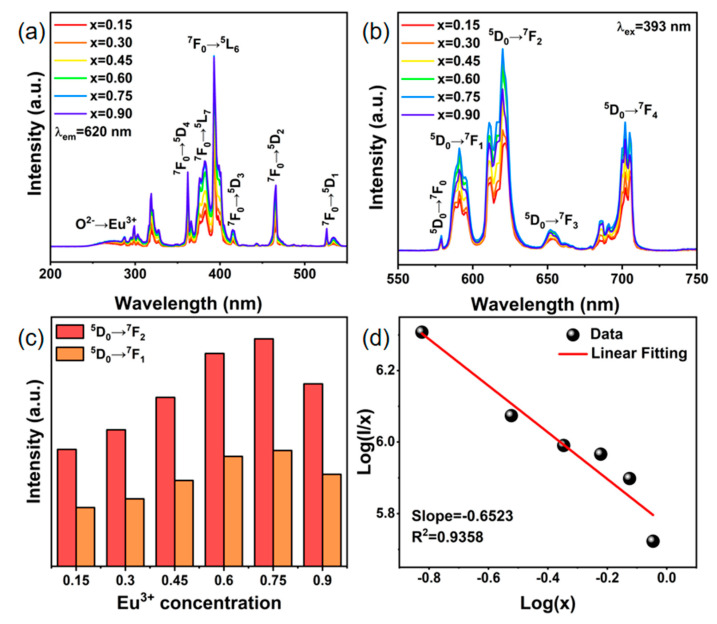
(**a**) PL excitation spectra (λ_em_ = 620 nm), (**b**) PL emission spectra (λ_ex_ = 393 nm), (**c**) changing trends of the transitions of ^5^D_0_ → ^7^F_1_ and ^5^D_0_ → ^7^F_2_ and (**d**) Linear fitting of log(x) vs. log(I/x) for the Na_3.6_Y_1.8−x_(PO_4_)_3_:xEu^3+^ phosphors.

The PL excitation and emission spectra of the Na_3.6_Y_1.8−x_(PO_4_)_3_:xEu^3+^ phosphors are presented in Figure 4a,b. The PL excitation spectra excited at 620 nm were measured in the range of 200–550 nm and made up of a broad excitation peak at 275 nm and several relatively narrow excitation peaks at 300–550 nm. The former is attributed to the O^2-^-Eu^3+^ charge transfer band (CTB), while the latter is the 4f-4f transition characteristic of Eu^3+^ [35]. The dominant excitation peak at 393 nm corresponds to the ^7^F_0_ → ^5^L_6_ transition of Eu^3+^, demonstrating that these samples can be effectively excited by near-ultraviolet light. Under excited at 393 nm, the PL spectra exhibit several emission peaks at 579 nm, 591 nm, 620 nm, 652 nm, and 702 nm, corresponding to the ^5^D_0_ → ^7^F_J_ (J = 0–4) transition of Eu^3+^, respectively [36,37]. Interestingly, the strongest emission peak occurs at 620 nm, with 702 nm following closely behind, which falls in the deep-red emission region (>650 nm). The emission intensity of the ^5^D_0_ → ^7^F_4_ transition is typically limited in the emission spectra of Eu^3+^-doped phosphor, while it is significant in these phosphors, suggesting that it can be used to promote plant development [38,39].

Furthermore, local coordination or the crystal field has a considerable impact on the electric dipole (ED) transition (^5^D_0_ → ^7^F_2_), yet neither has any effect on the magnetic dipole (MD) transition (^5^D_0_ → ^7^F_1_). When Eu^3+^ ions occupy non-inversion symmetry sites, the emission of ^5^D_0_ → ^7^F_2_ transition predominates, whereas the ^5^D_0_ → ^7^F_1_ transition acquires preponderance when Eu^3+^ ions are at the symmetry sites [40]. Therefore, the intriguing metric known as the asymmetry ratio (AR), which provides information about the local symmetry distortion around the Eu^3+^ ions in the host material, can be calculated by dividing the intensity of the ^5^D_0_ → ^7^F_2_ transition by the ^5^D_0_ → ^7^F_1_ transition [41]. As can be shown in Figure 4c, the PL intensity ratios for the ^5^D_0_ → ^7^F_2_ and ^5^D_0_ → ^7^F_1_ transitions remain close to 2 across different Eu^3+^ concentrations, indicating that Eu^3+^ ions occupy non-inversion symmetry sites. Moreover, since the valence electrons of Eu^3+^ are shielded from the outside 5s and 5p electrons, the surroundings in the crystal have less of a bearing on the 4f-4f transitions of Eu^3+^. Consequently, only the emission intensity varies on a regular basis, while the emission spectrum profiles and placements of these samples remain constant with Eu^3+^ concentration variations. When the Eu^3+^ concentration is x = 0.75, the emission intensity reaches the maximum and then decreases, which is caused by the concentration quenching effect. The quantum yields of the Na_3.6_Y_1.8−x_(PO_4_)_3_:0.75Eu^3+^ are displayed in Table 1, which can reach 81.26%.

Concentration quenching is a typical phenomenon resulting from energy migration between the Eu^3+^ activators as the concentration increases and the distance decreases [42]. To delve deeper into the specific concentration quenching mechanism, the relationship of log(*I*/*x*)~log(*x*) for Na_3.6_Y_1.8−x_(PO_4_)_3_:xEu^3+^ phosphors was analyzed using the Dexter formula [43]:

(4) $$\log\left(\frac{I}{x}\right)=C-\frac{\theta }{3}\log\left(x\right)$$

where *x* represents the dopant concentration of the activator, *I* is the corresponding emission intensity, *C* represents constants and *θ* values of 3, 6, 8, and 10 stand for the interactions involving nearest-neighbor ions, electric dipole–dipole, dipole–quadrupole, and quadrupole–quadrupole, respectively. As shown in Figure 4d, the slope (–*θ*/3) is found to be –0.6523, such that the calculated value of *θ* is 1.9569 and close to 3, indicating that nearest-neighbor ion interaction is the predominant concentration quenching mechanism in Na_3.6_Y_1.8−x_(PO_4_)_3_:xEu^3+^ phosphors.

**Figure 5:**
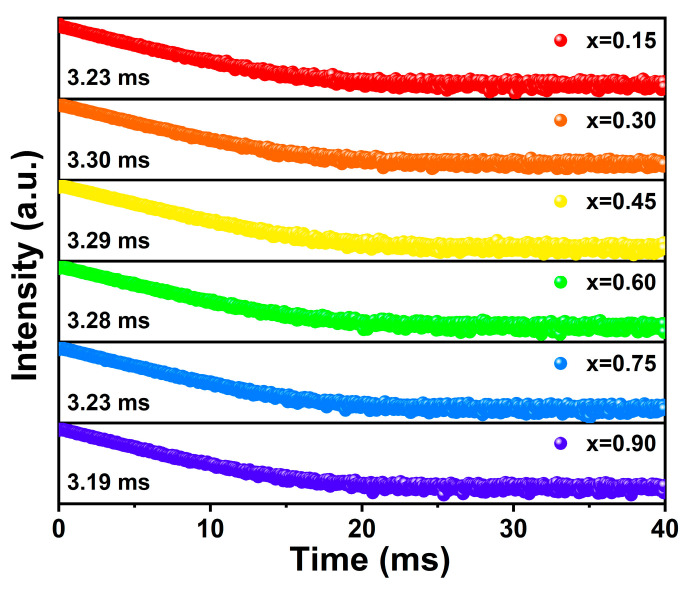
The decay curves (λ_ex_ = 393 nm, λ_em_ = 620 nm) of the Na_3.6_Y_1.8−x_(PO_4_)_3_:xEu^3+^ phosphors.

The investigation on decay behaviors constitutes a valuable approach towards elucidating the primary factors influencing emission intensity. The decay curves of Na_3.6_Y_1.8−x_(PO_4_)_3_:xEu^3+^ phosphors monitored at 620 nm upon excitation at 393 nm are depicted in Figure 5, amenable to fitting via the single exponential equation [44]:

(5) $$I(t)=Aexp\left(-\frac{t}{\tau }\right)+I_{0}$$

where *τ* represents decay time for exponential components, *I(t)* is the luminescence intensity at a time *t*, and *A* is fitting parameters. It is discernible that the luminescent lifetime exhibits an initial rise followed by a subsequent decline in tandem with the augmentation of Eu^3+^ concentration. This phenomenon can be explained by the interaction between Eu^3+^. When the content of Eu^3+^ is low, the interaction is negligible, resulting in a substantially reduced efficiency of energy transfer from the interior to the surface, thereby elongating the luminescence lifetime. Conversely, the escalation of Eu^3+^ content diminishes the distance amid luminescent centers, fostering the establishment of a network for resonance energy transfer, and expediting the non-radiative energy transfer from the inner part to the surface, consequently curtailing the lifetime.

### 3.5. Luminescence Properties of Li^+^/K^+^ Substitution Na_3.6_Y_1.05_(PO_4_)_3_:0.75Eu^3+^ Phosphors

**Figure 6:**
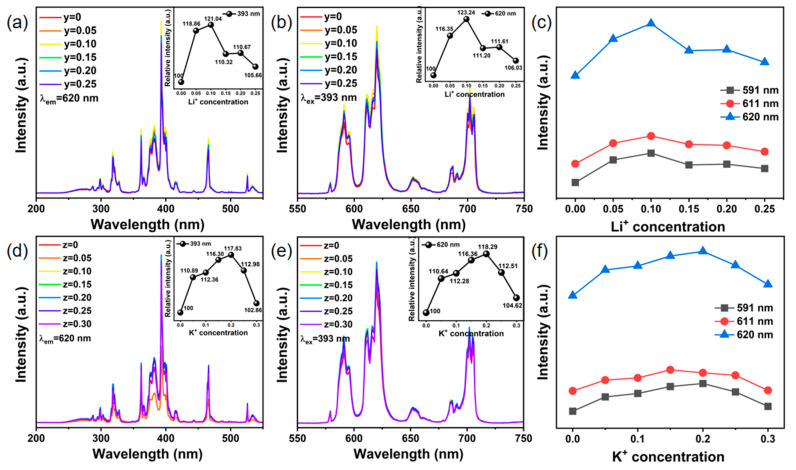
(**a**,**d**) PL excitation spectra (λ_em_ = 620 nm), (**b**,**e**) PL emission spectra (λ_ex_ = 393 nm) and (**c**,**f**) trend of three main emission peak intensities of Na_3.6-y_Li_y_Y_1.05_(PO_4_)_3_:0.75Eu^3+^ and Na_3.6-z_K_z_Y_1.05_(PO_4_)_3_:0.75Eu^3+^ phosphors.

The PL excitation (Figure 6a,d, λ_em_ = 620 nm) and emission (Figure 6b, e, λ_ex_ = 393 nm) spectra of the Na_3.6-y_Li_y_Y_1.05_(PO_4_)_3_:0.75Eu^3+^ and Na_3.6-z_K_z_Y_1.05_(PO_4_)_3_:0.75Eu^3+^ phosphors with varying proportions of Li^+^ and K^+^ substitution are depicted in Figure 6. It is evident that the PL excitation spectra still consist of a broad excitation band at 275 nm and multiple narrow excitation peaks, while the overall spectral shape remains constant as the Li^+^/K^+^ substitutions increase. Under the excitation of 393 nm, the emission characteristics of Eu^3+^ ions remain unaltered upon incremental Li^+^/K^+^ substitution, with consistent peak position and shape. Nevertheless, there is a noticeable trend in the 620 nm peak emission intensity of Eu^3+^, showing an initial increase followed by a decrease, with enhancements of 23.24% and 18.29% observed at y = 0.10 and z = 0.20, respectively. After testing, the quantum yields of this sample series are displayed in Table 1, with the highest values being 99.85% and 96.29%, respectively, demonstrating the outstanding luminescence performance of these samples. All these results indicate that alkali metal ion substitution can enhance the luminescence of Eu^3+^. Based on the previous XRD analysis, it can be inferred that due to the large variations in ionic radii, the gradual replacement of Na^+^ ions with Li^+^/K^+^ ions would inevitably result in changes in crystal structure and reduced symmetry. As Figure 6c,f demonstrate, the enhancement effect of the ^5^D_0_ → ^7^F_2_ transition peak is noticeable at 620 nm and 611 nm with Li^+^/K^+^ doping, whereas the enhancement effect of the ^5^D_0_ → ^7^F_1_ transition peak is less pronounced at 591 nm. Therefore, it is expected that the severe distortion and changed crystal field in the surrounding environment of the Eu^3+^ ions is anticipated to facilitate the occurrence of highly sensitive electron dipole transitions (^5^D_0_ → ^7^F_2_), which will further have a substantial influence on its emission intensity [45,46].

**Figure 7:**
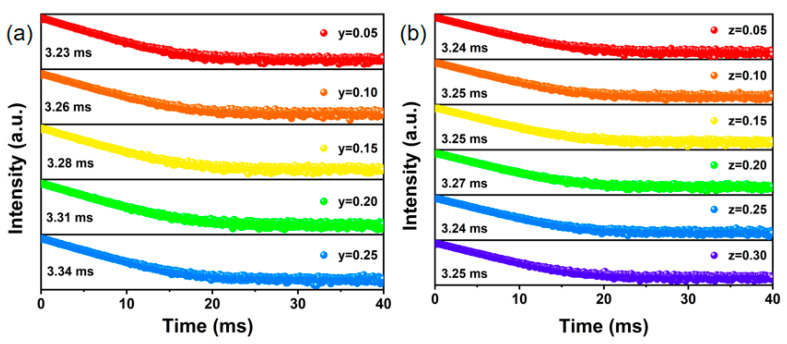
The decay curves (λ_ex_ = 393 nm, λ_em_ = 620 nm) of the (**a**) Na_3.6-y_Li_y_Y_1.05_(PO_4_)_3_:0.75Eu^3+^ and (**b**) Na_3.6-z_K_z_Y_1.05_(PO_4_)_3_:0.75Eu^3+^ phosphors.

To further investigate the impact of cation substitution on luminescence properties, the decay curves of the Na_3.6-y_Li_y_Y_1.05_(PO_4_)_3_:0.75Eu^3+^ and Na_3.6-z_K_z_Y_1.05_(PO_4_)_3_:0.75Eu^3+^ phosphors was tested, as illustrated in Figure 7. This directly reveals the correlation between Li^+^/K^+^ concentration and fluorescence lifetime. The luminescence lifetime was determined by fitting Equation (5), exhibiting a continuous increase corresponding to the rise in Li^+^/K^+^ content. This is because the addition of Li^+^/K^+^ ions produces lattice distortion and affects the energy level structure of Eu^3+^ in the phosphors, including the energy level spacing and energy level distribution. Consequently, this enhances the absorption of excitation light by the phosphors, leading to prolonged luminescence lifetime. Furthermore, with the increase in the content of K^+^ phosphor lifetimes appeared the phenomenon of the lower and higher values, showing that K^+^ doping also has an effect on the lifetime.

### 3.6. Thermal Stability, CIE Parameters and Application

**Figure 8:**
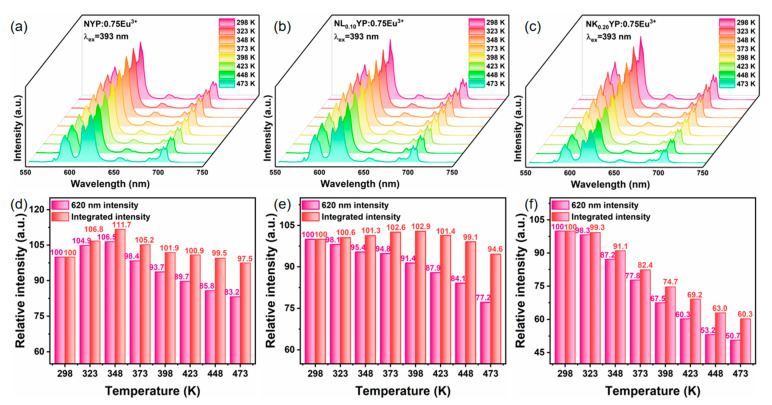
Emission spectra of the (**a**) Na_3.6_Y_1.05_(PO_4_)_3_:0.75Eu^3+^, (**b**) Na_3.5_Li_0.10_Y_1.05_(PO_4_)_3_:0.75Eu^3+^ and (**c**) Na_3.4_K_0.20_Y_1.05_(PO_4_)_3_:0.75Eu^3+^ phosphors with temperature-dependent curves at various operation temperatures; changing trends of the 620 nm intensity and integrated intensity of (**d**) Na_3.6_Y_1.05_(PO_4_)_3_:0.75Eu^3+^, (**e**) Na_3.5_Li_0.10_Y_1.05_(PO_4_)_3_:0.75Eu^3+^ and (**f**) Na_3.4_K_0.20_Y_1.05_(PO_4_)_3_:0.75Eu^3+^.

To further investigate the thermal stability of these phosphors, the PL spectra of Na_3.6_Y_1.05_(PO_4_)_3_:0.75Eu^3+^, Na_3.5_Li_0.10_Y_1.05_(PO_4_)_3_:0.75Eu^3+^ and Na_3.4_K_0.20_Y_1.05_(PO_4_)_3_:0.75Eu^3+^ phosphors were examined across a temperature range of 298–473 K, as depicted in Figure 8a–c. The histograms which show the change trends of integrated intensity and the strongest peak intensity at 620 nm are shown in Figure 8d–f. It can be seen that as the temperature increased, the integrated intensity of Na_3.6_Y_1.05_(PO_4_)_3_:0.75Eu^3+^ and Na_3.5_Li_0.10_Y_1.05_(PO_4_)_3_:0.75Eu^3+^ presents a trend of first increasing and then decreasing, showing a non-thermal quenching effect, and reached the maximum of 111.7% and 102.9% at 348 and 398 K, respectively. However, the integrated strength of Na_3.4_K_0.20_Y_1.05_(PO_4_)_3_:0.75Eu^3+^ phosphor does not have such a non-thermal quenching phenomenon. Finally, at 473 K, the integrated emission intensities of these samples were found to be 97.5%, 94.6% and 60.3% of that at room temperature. Unexpectedly, the emission intensity of the strongest peak at 620 nm decreased more dramatically, and non-thermal quenching was not observed in the alkali metal ion-substituted samples. As seen in Figure S4, the variations in the luminescent emission at varying temperatures were produced by normalizing the intensity of the peak at 620 nm. As the temperature rises, it is evident that the emission intensity of other emission peaks exhibits an increasing trend in comparison to the 620 nm peak, so that the integral intensity shows a non-thermal quenching phenomenon. Even though the luminescence intensity of Na_3.5_Li_0.1_Y_1.05_(PO_4_)_3_:0.75Eu^3+^ phosphor decreased more substantially, it showed a 23.24% increase upon Li^+^ ion substitution. Consequently, the luminous intensity of Na_3.5_Li_0.1_Y_1.05_(PO_4_)_3_:0.75Eu^3+^ still surpasses that of Na_3.6_Y_1.05_(PO_4_)_3_:0.75Eu^3+^. To explore the correlation between luminescence intensity and temperature, the activation energy (*ΔEa*) was determined based on the intensity of the most prominent peak using the Arrhenius equation [36]:

(6) $$I_{T}={I_{0}\left[1+C\exp\left(-\frac{\Delta Ea}{kT}\right)\right]}^{-1}$$

where *I_T_* and *I_0_* are the emission intensity at temperatures *T* and 298 K, respectively, *C* is the constant, and *k* is the Boltzmann constant. By measuring the emission intensity at different temperatures, the ln[(*I_0_*/*I_T_*)−1] was used as the x value and 1/*kT* as the y value for linear fitting, as shown in Figure S5. The Δ*Ea* for Na_3.6_Y_1.05_(PO_4_)_3_:0.75Eu^3+^, Na_3.5_Li_0.1_Y_1.05_(PO_4_)_3_:0.75Eu^3+^ and Na_3.4_K_0.20_Y_1.05_(PO_4_)_3_:0.75Eu^3+^ phosphors were determined to be 0.3728, 0.2241 and 0.2175 eV, underscoring the excellent thermal stability of these phosphors, which is consistent with the experimental results. During the crossover process involving the thermal quenching of Eu^3+^ luminescence, electrons must surmount a specific energy barrier (i.e., Δ*Ea*); thus, a higher Δ*Ea* value hinders the crossover process and enhances the thermal stability of the phosphor [47]. In the use of agricultural film and plant growth LEDs, the temperature from inside is typically higher than room temperature, and consequently, the non-thermal quenching effect and superior thermal stability of these phosphors are advantageous for practical applications.

**Figure 9:**
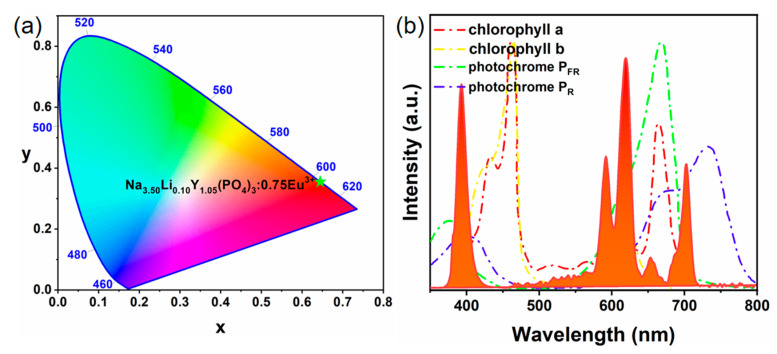
(**a**) CIE coordinates and (**b**) PL emission spectra (and the absorption curves of the plant pigments of chlorophyll a/b, photochrome P_R_ and P_FR_) of Na_3.5_Li_0.10_Y_1.05_(PO_4_)_3_:0.75Eu^3+^ phosphor.

Since Li^+^/K^+^ substitution only changes the emission intensity of Eu^3+^ without affecting its shape, the representative sample Na_3.5_Li_0.10_Y_1.05_(PO_4_)_3_:0.75Eu^3+^ was selected to explore its application in plant growth. The CIE (Commission International del’Eclairage) chromaticity color diagram for Na_3.5_Li_0.10_Y_1.05_(PO_4_)_3_:0.75Eu^3+^ phosphor is shown in Figure 9a, which shows that this phosphor can achieve orange-red light and the CIE coordinates are located at (0.6448, 0.3549). A 395 nm n-UV chip is coated with as-prepared Na_3.5_Li_0.10_Y_1.05_(PO_4_)_3_:0.75Eu^3+^ phosphor to produce an actual red LED, the emission spectrum of which is shown in Figure 9b. It can be seen that the emission spectrum of the LED is in good agreement with the absorption spectrum of plant pigments because Eu^3+^ ions in the phosphor are located at the ^5^D_0_ → ^7^F_4_ transition emission peak at 705 nm. Therefore, the as-prepared phosphors have the potential to be used in plant growth applications.

## 4. Conclusions

In this study, a series of Eu^3+^-doped and Li^+^/K^+^ partially substituted Na_3.6_Y_1.8_(PO_4_)_3_ red-emitting phosphors have been successfully prepared. A few interesting findings have been summarized, as follows:

(i) Confirmed by XRD and EDS techniques, the calcination temperature was found to be key for the formation of the as-obtained Na_3.6_Y_1.8_(PO_4_)_3_:Eu^3+^ phosphors, and thus, various temperatures for calcinations were examined for better control under the reported reaction conditions.
(ii) By a 393nm n-UV excitation, the as-prepared Na_3.6_Y_1.8−x_(PO_4_)_3_:xEu^3+^ phosphors exhibited red emission with the highest peak located at ~620 nm and a maximum quantum yield of 81.26%. Interestingly, the intensity of the ^5^D_0_ → ^7^F_4_ emission peak at ~705 nm of the phosphors is significant, making the emission spectra more accurately match the absorption of plant pigments. The representative Na_3.6_Y_1.05_(PO_4_)_3_:0.75Eu^3+^ sample exhibits a non-thermal quenching effect along with excellent thermal stability (97.5%@473 K).
(iii) XRD and SEM analysis revealed that the crystal structure and morphology of the Na_3.6_Y_1.05_(PO_4_)_3_:0.75Eu^3+^ sample remained unaltered in response to a tiny amount of Li^+^/K^+^ doping. By partial substitution of Na^+^ by Li^+^/K^+^ ions to change the crystal field and reduce the symmetry around Eu^3+^, the luminescence intensity of Na_3.6_Y_1.05_(PO_4_)_3_:0.75Eu^3+^ phosphor is significantly increased by 23.24% and 18.29%, and the highest quantum yields reach 99.85% and 96.29%, respectively. Furthermore, a red-emission plant growth LED model was established using a 395 nm n-UV chip coated with Na_3.5_Li_0.10_Y_1.05_(PO_4_)_3_:0.75Eu^3+^ phosphors, in which the emission spectrum could greatly comply with the P_R_ absorption spectrum of plants.

In general, this study has demonstrated that the substitution of alkali metal ions can significantly enhance the luminescence intensity of Eu^3+^-doped phosphors, and show strong potential serving as red phosphors for plant growth, anti-counterfeiting and biological imaging, because of their excellent optical performance, thermal stability, and compatibility.

## Figures and Tables

**Table 1:** Quantum yields of the Na_3.6-y_Li_y_Y_1.05_(PO_4_)_3_:0.75Eu^3+^ and Na_3.6-z_K_z_Y_1.05_(PO_4_)_3_:0.75Eu^3+^ phosphors excited at 393 nm.

y	0	0.05	0.10	0.15	0.20	0.25
QY (%)	81.26	84.90	99.85	92.91	74.87	81.60
z	0	0.05	0.10	0.15	0.20	0.25
QY (%)	81.26	76.35	84.79	92.04	96.29	90.57

## Data Availability

Data is contained within the article.

## Supplementary Materials

The following supporting information can be downloaded at: https://www.mdpi.com/article/10.3390/nano14211721/s1, Figure S1. Powder XRD patterns of (a) Na_3.6_Y_1.8_(PO_4_)_3_:Eu^3+^ phosphors synthesized by calcination at 1100–1500 °C, (b) Na_3.6_Y_1.8_-x(PO_4_)_3_:xEu^3+^ (0 ≤ x ≤ 0.90). Figure S2. SEM image of (a) host, (b) Na_3.5_Li_0.1_Y_1.05_(PO_4_)_3_:0.75Eu^3+^ and (c) Na_3.6_K_0.2_Y_1.05_(PO_4_)_3_:0.75Eu^3+^ samples. Figure S3. TEM and EDS elemental mappings (K, Na, Y, P, O and Eu) images of Na_3.5_Li_0.1_Y_1.05_(PO_4_)_3_:0.75Eu^3+^ and Na_3.6_K_0.2_Y_1.05_(PO_4_)_3_:0.75Eu^3+^ samples. Figure S4. Normalized emission spectra of (a) Na_3.6_Y_1.05_(PO_4_)_3_:0.75Eu^3+^, (b) Na_3.5_Li_0.1_Y_1.05_(PO_4_)_3_:0.75Eu^3+^ and (c) Na_3.6_K_0.2_Y_1.05_(PO_4_)_3_:0.75Eu^3+^ samples. Figure S5. Plot of Ln(I0/IT-1) versus 1/kT of the (a) Na_3.6_Y_1.05_(PO_4_)_3_:0.75Eu^3+^, (b) Na_3.5_Li_0.1_Y_1.05_(PO_4_)_3_:0.75Eu^3+^ and (c) Na_3.6_K_0.2_Y_1.05_(PO_4_)_3_:0.75Eu^3+^ samples.
