# Supplementary material for: Preparation and Luminescence Property Study of Red-Emitting Na3.6Y1.8(PO4)3:Eu3+,Li+/K+ Phosphors with Excellent Thermal Stability for Light-Conversion Application
Source: Nanomaterials (Basel). 2024 Oct 29;14(21):1721. doi: 10.3390/nano14211721 (PMC11547445; doi:10.3390/nano14211721)
Supplement: Supplementary file 1 [file nanomaterials-14-01721-s001.zip › nanomaterials-3243339-supplementary.pdf]

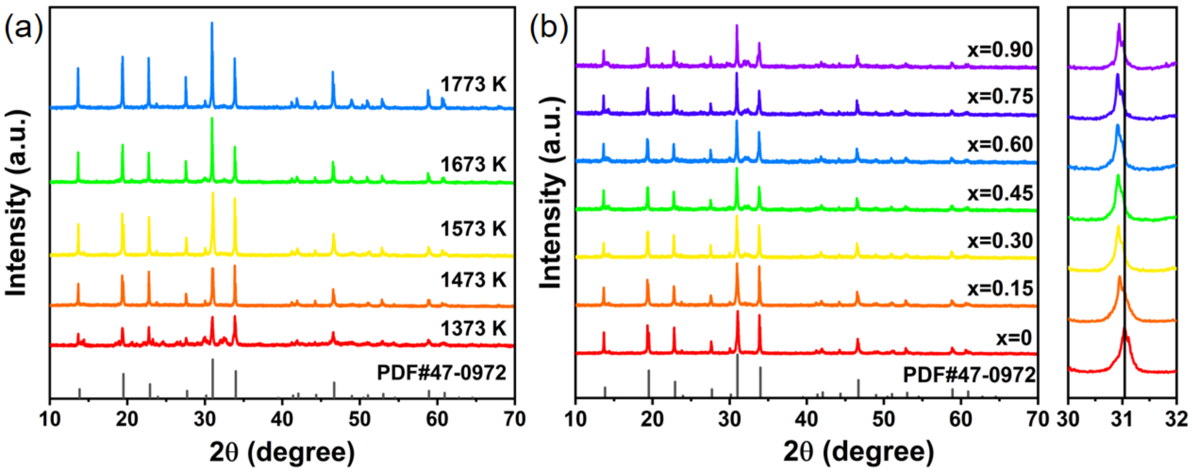

Figure S1. Powder XRD patterns of (a) $Na_{3.6}Y_{1.8}(PO_4)_3:Eu^{3+}$ phosphors synthesized by calcination at 1100-1500 °C, (b) $Na_{3.6}Y_{1.8-x}(PO_4)_3:xEu^{3+}$ ($0 \leq x \leq 0.90$).

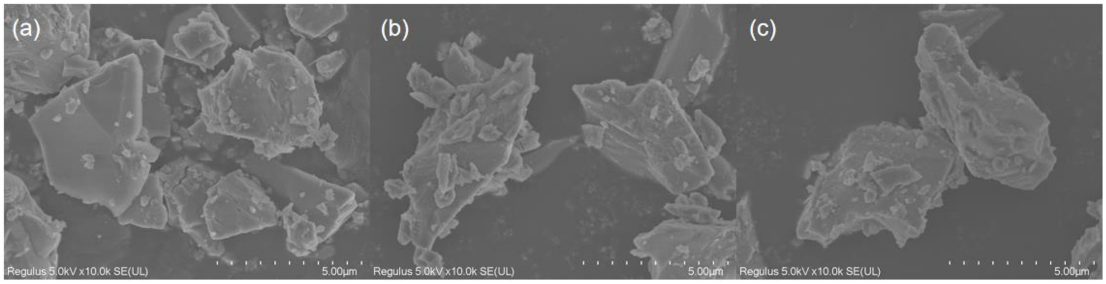

Figure S2. SEM image of (a) host, (b) $Na_{3.5}Li_{0.1}Y_{1.05}(PO_4)_3:0.75Eu^{3+}$ and (c) $Na_{3.6}K_{0.2}Y_{1.05}(PO_4)_3:0.75Eu^{3+}$ samples.

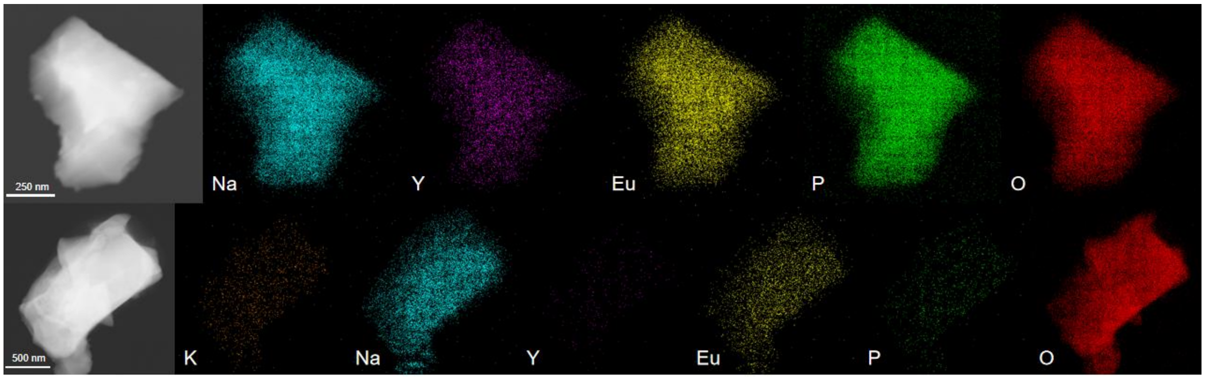

Figure S3. TEM and EDS elemental mappings (K, Na, Y, P, O and Eu) images of $Na_{3.5}Li_{0.1}Y_{1.05}(PO_4)_3:0.75Eu^{3+}$ and $Na_{3.6}K_{0.2}Y_{1.05}(PO_4)_3:0.75Eu^{3+}$ samples.

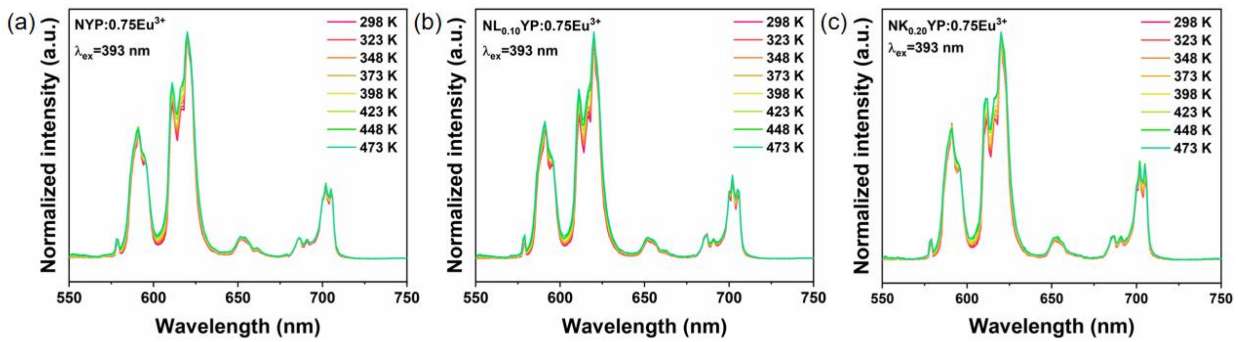

Figure S4. Normalized emission spectra of (a) $Na_{3.6}Y_{1.05}(PO_4)_3$:0.75Eu^{3+}, (b) $Na_{3.5}Li_{0.1}Y_{1.05}(PO_4)_3$:0.75$Eu^{3+}$ and (c) $Na_{3.6}K_{0.2}Y_{1.05}(PO_4)_3$:0.75$Eu^{3+}$ samples.

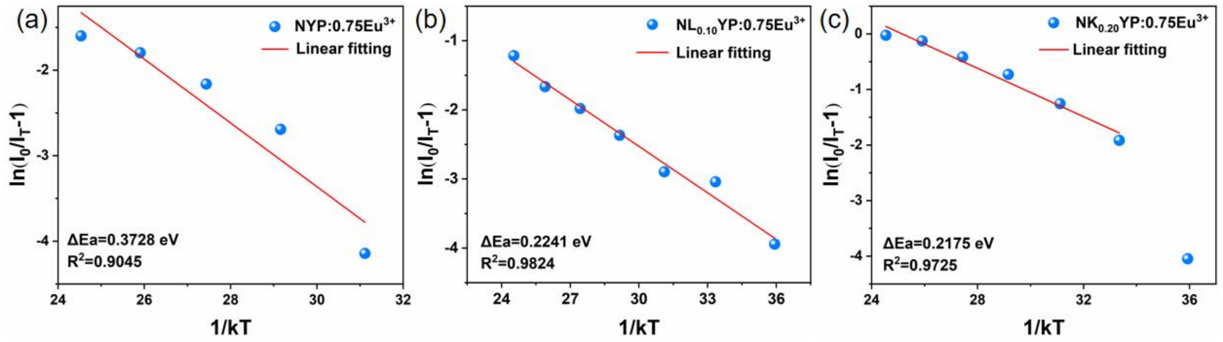

Figure S5. Plot of Ln(I_0/I_T-1) versus 1/kT of the (a) $Na_{3.6}Y_{1.05}(PO_4)_3$:0.75Eu^{3+}, (b) $Na_{3.5}Li_{0.1}Y_{1.05}(PO_4)_3$:0.75$Eu^{3+}$ and (c) $Na_{3.6}K_{0.2}Y_{1.05}(PO_4)_3$:0.75$Eu^{3+}$ samples.
